# Targeting the BAG2/CHIP axis promotes gastric cancer apoptosis by blocking apoptosome assembly

**DOI:** 10.3389/fimmu.2025.1578416

**Published:** 2025-07-18

**Authors:** Qian Liu, Qingjie Chen, Hong Wei, Baoyuan Tang, Binbin Tian, Zhijian Ma, Qianlin Gu, Xiaolu Su, Yuman Dong, Wengui Shi, Changjiang Luo

**Affiliations:** Department of General Surgery, The Second Hospital of Lanzhou University, Lanzhou, China

**Keywords:** BAG2, gastric cancer, apoptosis, apoptosome, FIIN-2

## Abstract

Apoptosis has been shown to play an important role in the treatment of gastric cancer, and BCL2-associated athanogene 2(BAG2) has been found to be able to inhibit apoptosis by interacting with multiple apoptosis regulators. In this study, we demonstrate that BAG2 functions as an independent prognostic factor, correlating with unfavorable clinical outcomes in patients with gastric cancer (GC). We demonstrate that BAG2 upregulation inhibited apoptosis and increased proliferation, migration, and invasion of GC cells, whereas the opposite results were obtained in BAG2-deficient GC cells. Mechanistically, BAG2 interacts with the c-terminus of HSP70-interacting protein(CHIP) to inhibit the ubiquitination degradation of Heat shock protein70(HSP70) and increase the binding of HSP70 to apoptotic protease-activating factor 1(Apaf1). The reduced ubiquitination degradation of HSP70 reduces the release of mitochondrial cytochrome C (Cytc), which ultimately inhibits the formation of apoptotic bodies assembled by Cytc and Apaf1. The above effects of BAG2 inhibit the formation of Cytc and Apaf1-assembled apoptotic bodies. Furthermore, we screened FIIN-2, an inhibitor of the BAG2 complex, which effectively halts the malignant development of GC triggered by reduced apoptosis by blocking BAG-CHIP binding. In conclusion, this study highlights BAG2’s key role in regulating apoptosis and confirms FIIN-2’s effectiveness in GC-targeted therapy.

## 1 Introduction

Gastric cancer is the fifth most prevalent malignant tumor worldwide and the fourth leading cause of malignancy-related death. Despite a decline in the overall incidence of gastric cancer in recent years, a marked increase has been observed in the prevalence of early-onset cancers. A multifaceted etiology contributes to the development of this malignancy, with well-established risk factors including Helicobacter pylori infection, excess body fat, smoking, and a high-salt diet. Other possible risk factors include Epstein–Barr virus (EBV) infection, autoimmune gastritis, and Ménétrier’s disease (1, 2). As most patients with gastric cancer are often diagnosed at an advanced stage, the mortality rate is high, and the prognosis is usually poor, with a low long-term survival rate even after comprehensive treatment (3). There is mounting evidence that gastric cancer cells exhibit elevated levels of aberrant gene expression or mutational load (4, 5). Consequently, further elucidation of the underlying molecular mechanisms that promote apoptosis in gastric cancer cells will facilitate the development of biomarker-driven therapeutic targets for individual gastric cancer patients.

In the context of cancer, apoptosis is a prevalent tumor suppressor mechanism, a property that plays a pivotal role in cancer therapy. However, cancer cells frequently devise strategies to evade apoptosis or defects in apoptotic pathways, thereby enabling their escape and survival (6, 7). One potential approach involves the reactivation of apoptotic signaling pathways in cancer cells, which could lead to the targeting and elimination of these cells, a reduction in tumor load, and an enhancement in the efficacy of chemotherapy and radiotherapy. A range of approaches have been developed to promote apoptosis, including the development of apoptosis-inducing drugs, gene therapies, immunotherapies, and combination therapeutic strategies. These approaches aim to restore the normal apoptotic mechanism of cancer cells, inhibit tumor angiogenesis, and enhance the ability of the immune system to kill cancer cells (8–10). By these means, the growth and spread of tumors can be effectively controlled, and the efficacy of cancer treatments can be improved. Bcl-2 Associated Athanogene 2 (BAG2) is a protein that is widely expressed in cells and plays a key role in a variety of biological processes, especially in the regulation of apoptosis. BAG2 hinders apoptotic signaling by interacting with a variety of proteins, mainly by inhibiting the activation of cysteine asparaginase (Caspase). Consequently, it plays a role in protecting cells from apoptosis (11, 12). The study of the relationship between BAG2 and apoptosis contributes to an in-depth understanding of the molecular mechanisms of cell survival and death and may provide new targets and strategies for the treatment of related diseases.

During the process of apoptosis, the central roles of apoptotic protease-activating factor 1 (Apaf1) and cytochrome C (Cytc) have been well-documented. Specifically, Apaf1, through its structural properties, is able to bind to Cytc released from the mitochondria. In the presence of ATP/dATP, Apaf1 promotes the assembly of apoptotic bodies, which in turn activates the precursor caspase-9 and initiates the caspase cascade reaction that ultimately leads to apoptosis (13–15). The key to this process lies in the synergistic interaction between Apaf1 and Cytc, which together constitute the apoptosome and regulate the cell fate transition. Furthermore, heat shock protein 70 (HSP70) exerts a certain degree of inhibition on apoptosis by impeding apoptosome formation and caspase activation through its interaction with Apaf1 (16, 17). Of particular significance is the direct binding of HSP70 to Cytc, thereby preventing its release from the mitochondria into the cytoplasm and further hindering apoptosome assembly and caspase activation (17). This process is critically important for the synergy between Apaf1 and Cytc, which together constitute the apoptosome and regulate the cell fate transition and caspase activation. This dual regulatory mechanism not only reveals the multiple roles of HSP70 in apoptosis but also provides new perspectives on its potential applications in disease therapy. Consequently, an in-depth examination of the roles of Apaf1 and Cytc in apoptosis, along with their regulatory mechanisms involving HSP70, is imperative to comprehending the molecular underpinnings of apoptosis and formulating targeted therapeutic strategies.

In this study, an analysis of The Cancer Genome Atlas (TCGA) database was conducted to identify dysregulated genes associated with apoptosis-associated gastric cancer. It was determined that BAG2 is highly expressed in gastric cancer. Furthermore, our findings indicate that enforced BAG2 expression enhances the proliferation and metastasis of GC cells in both *in vitro* and *in vivo* models. Mechanistically, our data suggest that BAG2 prevents the ubiquitination and degradation of HSP70 by binding to CHIP, followed by an increase in the binding of HSP70 to Apaf1, which inhibits the formation of apoptosome. Furthermore, HSP70 blocked cystic-mediated caspase activation, thereby preventing apoptosis. In summary, our study emphasized the importance of BAG2 in GC patients and revealed a novel mechanism of apoptosis inhibition by BAG2. Furthermore, we successfully screened the potential of FIIN-2, a complex inhibitor, for the treatment of gastric cancer.

The present study is pioneering in its identification of BAG2 as a pivotal regulator of the CHIP-HSP70 axis and a modulator of apoptosis in gastric cancer, thereby establishing it as an independent adverse prognostic factor. This research extends the scope of studies that have focused exclusively on the CHIP-HSP70 interaction by introducing BAG2 as a critical upstream regulatory switch. A substantial body of research has been dedicated to elucidating the mechanisms by which CHIP ubiquitinates and degrades HSP70, or the functions of HSP70 itself. The present study reveals that BAG2 acts as a negative regulator of CHIP, stabilizing HSP70 by inhibiting CHIP’s E3 ligase activity. This represents a novel upstream, positive regulatory pathway for HSP70 stability.

## 2 Materials and methods

### 2.1 Clinical specimens of human GC

All specimens were obtained with approval from the Ethics Review Committee of the Second Hospital of Lanzhou University. Informed consent was obtained from all participants. A total of 152 pairs of GC and adjacent normal tissue specimens were collected, and TMAs were prepared for IHC staining after formalin fixation and paraffin embedding.

### 2.2 Cell lines and cell culture

Human GC cell lines were obtained from the Institute of Basic Medical Sciences, Chinese Academy of Medical Sciences (Beijing, China): NCI-N87, HGC-27, MKN-45, and AGS. The HEK-293T cell line was obtained from ATCC, and the SNU-216 human GC cell line was obtained from the КОrean Cell Line Bank. All cell lines were tested for mycoplasma contamination and validated via short tandem repeat DNA fingerprinting using the commercially available EX20 Kit from AGCU. NCI-N87 and SNU-216 cells were cultured in RPMI 1640 medium with 10% fetal bovine serum (FBS), whereas the remaining cell lines were cultured in Dulbecco’s modified Eagle’s medium (DMEM) supplemented with 10% FBS. All cell lines were cultured under humid conditions at 37°C with 5% CO_2_.

### 2.3 Plasmids and RNA interference

The expression vectors were generated using the Gibson Assembly cloning method. The cDNA sequences of BAG2, CHIP, and Apaf-1 were inserted into either the pRK5-FLAG or pRK5-3HA vectors for transient expression. Furthermore, the cDNA sequences of BAG2 and CHIP were added to the pRK5-NOTAG vector. CHIP gene mutants (91–96 aa, 131–135 aa, 91–96 aa, and 131–135 aa) were produced using site-directed mutagenesis and then inserted into the pRK5-FLAG vector. All constructs were confirmed through full-length sequencing.

### 2.4 BAG2-specific sgRNAs and LV-sgRNAs transfection

BAG2 was knocked out and overexpressed using CRISPR/Cas9 gene editing and gene overexpression technologies. CRISPR/Cas9 and overexpression lentiviruses were purchased from GENE (Shanghai, China). An appropriate amount of lentivirus was added for viral infection, and the medium was replaced with fresh medium containing 10% FBS after 18-24h of infection. Puromycin was added for selection after 48h, and western blotting was performed to verify the efficiency of BAG2-КО and overexpression.

### 2.5 IHC

In GC tissues, IHC staining was performed to detect BAG2, HSP70, caspase-3, caspase-9, and Ki67. GC tissues were de-paraffinized and rehydrated, followed by antigen retrieval. Two pathologists from Lanzhou University Second Hospital assessed the staining intensity (0, no staining; 1, weak staining; 2, moderate staining; 3, strong staining) and the percentage of positive cells (0-100%). The tissue score (IHC score) was calculated by multiplying the staining intensity by the percentage of positive cells. An IgG antibody was used as the isotype control. The primary antibodies used were anti-BAG2 (Sigma, diluted 1:200), anti-HSP70 (Abcam, 1:200), anti-caspase-3 (Abcam, 1:200), anti-caspase-9 (Abcam, 1:200), and anti-Ki67 (Abcam, 1:300).

### 2.6 RT-PCR

Total RNA was extracted from the collected cells using Trizol reagent and reverse-transcribed into cDNA according to the manufacturer’s instructions. Quantitative PCR amplification and product detection were performed using SYBR Green dye (Takara) and 10 μM forward and reverse primers on a LightCycler instrument. The primer sequences used for qRT-PCR are listed in **Table 1**.

**Table 1:** Primer sequences used for qRT-PCR.

Gene	Primer(5’-3’)
BAG2	FW: ATCAACGCTAAAGCCAACGAG
	RV: CGTCACTGATCTGCCTCATGT
GAPDH	FW: GAAGGCTGGGGCTCATTT
	RV: CAGGAGGCATTGCTGATGAT

### 2.7 Immunoblotting

The cells were lysed using RIPA buffer and centrifuged at 12,000 ×g for 10 min. Protein lysates were separated via SDS/PAGE, transferred to PVDF membranes, and incubated overnight at 4°C with rabbit anti-BAG2 (1:1000; Abcam), rabbit anti-BAX (1:1000; Proteintech), rabbit anti-BCL2 (1:1000; Proteintech), rabbit anti-caspase-3 (1:1000; Abcam), rabbit anti-cleaved caspase-3 (1:1000; Abcam), rabbit anti-caspase-9 (1:1000; Abcam), rabbit anti-CHIP (1:1000; Abcam), rabbit anti-HSP70 (1:1000; Abcam), rabbit anti-Apaf1 (1:1000; Abcam), rabbit anti-Cytc (1:1000; Abcam), mouse anti-HA (1:1000; Invitrogen), rabbit anti-FLAG (1:1000; Abcam), and mouse anti-GAPDH (1:1000; Proteintech) antibodies. After removing the primary antibodies, the membranes were incubated with goat anti-rabbit or anti-mouse IgG secondary antibodies (1:10,000; Proteintech) and visualized using an enhanced chemiluminescence method.

### 2.8 MTT assay

Approximately 3000 cells were seeded per well in a 96-well plate. Cell viability was measured via OD490 after 2h of reaction with Cell-Titre 96^®^ AQueous One Solution reagent (G3582, Promega, USA).

### 2.9 Colony formation assay

1000 cells were seeded in 35-mm dishes and, after 10 days, fixed with 4% paraformaldehyde, stained with 0.05% crystal violet, and counted using ImageJ V1.53c software.

### 2.10 Cell scratch assay

Cell migration was evaluated using a scratch assay. Log-phase cells (3 × 10^4^) were seeded in 96-well plates and cultured until 90% confluence was achieved. Subsequently, three parallel linear wounds were generated using a micro-scratch tester. The migration rate was evaluated after an additional 8h and 24h culture periods. Three representative photographs of scratches per plate were captured using a microscope and analyzed.

### 2.11 Transwell invasion assay

A matrix-coated Transwell invasion assay was performed to measure invasive ability. Briefly, 1 × 10^5^ cells in the logarithmic phase were suspended in 500 µL of cell culture medium and added to the top chamber of a matrix-coated 24-well plate (Corning, Cambridge, MA, USA). The cells were then incubated for 48 h in a 37°C cell incubator. The membranes were fixed and stained with Giemsa solution for 5 min. After removing the remaining cells from the membrane, the number of cells below the membrane was counted.

### 2.12 Xenograft studies

All animals received humane care according to the criteria outlined in the “Guide for the Care and Use of Laboratory Animals” prepared by the National Academy of Sciences and published by the National Institute of Health. MKN-45/luc tumor xenograft models were established in athymic nude mice. The tumors were measured using a caliper three times per week. The animals were sacrificed, and IHC staining was performed on the tumors after 21 days. At the end of the experiments, the animals were sacrificed, and subcutaneous tumors were harvested. The animals were manipulated and housed according to protocols approved by the Animal Ethics Committee of Lanzhou University Second Hospital(D2024-630).

### 2.13 Flow cytometry

The cells were digested with trypsin and washed with PBS. After adjusting the cell concentration to 2 × 10^5^ cells/tube, cells were resuspended in 100 µL of binding buffer. Each sample was treated with 5 µL of Annexin V-FITC or APC and 10 µL PI or 7AAD (YESEN), incubated at room temperature for 15 min in the dark, and 400 µL binding buffer was added to each sample. The samples were then analyzed using flow cytometry.

### 2.14 TUNEL staining

TUNEL assays tested the apoptosis of GC cells according to the instruction book. Briefly, after 36 h, cells were fixed with 4% paraformaldehyde. The cells were washed and permeabilized with TritonX-100 for 10 min. Then, cells were treated with TUNEL staining (YESEN, China) and observed under a microscope.

### 2.15 Affinity purification and mass spectrometry analysis

AGS stable lines expressing FLAG-epitope-tagged BAG2 were seeded in 10 cm dishes and treated as indicated in the figure legends. Cells were lysed with lysis buffer [50 mM Tris-HCl (pH 7.4), 150 mM NaCl, 1 mM EDTA, and 0.2% TritonX-100] containing protease inhibitors. Lysates were centrifuged at 13,800 ×g for 10 min at 4°C. The supernatants were incubated with 50 μL prewashed anti-Flag M2 affinity gel agarose (50% slurry, Sigma) for 2h on a rotary shaker at 4°C. Immunoprecipitates were collected through centrifugation at 1500 ×g for 2 min at 4°C, washed thrice with 1 mL of cold lysis buffer, and eluted by adding 0.1 M glycine HCl (pH = 3.5).

### 2.16 Confocal microscopy analysis

Cells cultured in 35 mm glass-bottomed microwell dishes were fixed with 4% paraformaldehyde for 10 min and permeabilized with 0.1% TritonX-100. After that, cells were incubated with 3% FBS for 1 h at room temperature and then with primary antibodies overnight at 4°C, washed thrice in PBS and further incubated with the appropriate fluorescent-labeled secondary antibodies. Nuclei were counterstained with 4, 6-diamidino-2-phenylindole (DAPI) before mounting. Confocal fluorescence images were captured using a Zeiss LSM 880 laser microscope (×63 oil objective; Plan-Apochrom 1.4). The positivity of Flag-BAG2 or BAG2, CHIP, and HSP70 in the cells was detected via BAG2, CHIP, and HSP70 immunofluorescence and quantified using ImageJ V1.53c software. The mean fluorescence intensity (MFI) of BAG2, CHIP, and HSP70 in the nucleus was quantified, and cells with an MFI > 10 were identified as positive.

### 2.17 Electron microscopy examination

The HGC-27 cells were washed with PBS and digested with 0.25% trypsin; the cell suspension was collected and centrifuged at 2500 r, 4°C for 10 min. The supernatant was removed, 2.5% glutaraldehyde solution with a volume of 5–10 times the volume of cell clusters was added, and the mixture was incubated at room temperature for 15 min, centrifuged at 5000 r for 10 min, and refrigerated at 4°C overnight. Cell slices were prepared, and transmission electron microscopy was used to capture the apoptotic bodies.

### 2.18 Co-immunoprecipitation assay

Cells seeded in 10 cm dishes were lysed with lysis buffer [50 mM Tris-HCl (pH 7.4), 150 mM NaCl, 1 mM EDTA, and 0.2% Triton X-100] containing protease inhibitors. After 10 min on ice, lysates were centrifuged at 12,000 ×g for 10 min at 4°C. The supernatants were incubated with 25 μL prewashed anti-Flag M2 affinity gel agarose (50% slurry, Sigma) for 2 h on a rotary shaker at 4°C. Immunoprecipitants were collected via centrifugation at 1500 ×g for 2 min at 4°C, washed thrice with 1 mL of cold lysis buffer, and eluted by adding 0.1 M glycine HCl (pH 3.5). Finally, the protein samples were analyzed via SDS–PAGE and immunoblotted with antibodies against HA and FLAG.

### 2.19 Protein-protein docking

The binding mode of BAG2(AF-O95816-F1-model-v4) and CHIP (ID: 8F14) was calculated using the protein docking websites Cluspro. BAG2 and CHIP (ID: 8F14) were designated as the receptor and ligand proteins, respectively, with no specific amino acids predefined as interfaces or interacting residues. The docking model with the highest ClusPro score was selected for further validation.

### 2.20 Virtual screening of inhibitor targeting BAG2-CHIP interaction

A molecular docking analysis was conducted on the two binding pockets (site1 and site3) of the CHIP protein, resulting in the acquisition of affinity data and binding patterns for 17,675 compounds from the compound library. The relationship between the affinity scores of these compounds and their molecular weights is demonstrated in the **Supplementary Figure**. The compounds’ affinities for the target range from -12.5 kcal/mol to -2 kcal/mol (where lower values indicate stronger affinity), and their molecular weights range from 45 to 2000 Da. A correlation has been observed between the strength of affinity and the molecular weight of these compounds. To analyze the interaction patterns between small-molecule compounds and the target, we employed the protein-ligand interface fingerprint (PLIF) method to analyze their interaction sites and force types with CHIP. The compounds were then subjected to a screening process, with the parameters including a molecular weight of less than 1500 Da and an affinity score S of less than -9 kcal/mol. The structural diversity of the screened compounds was analyzed using StarDrop, after which duplicates and fatty chain molecules were removed, leaving a total of 144 molecules. These were subsequently analyzed through common substructure clustering, with similarity set to 0.3, yielding 28 distinct categories. A comprehensive analysis of the affinity values, molecular weights, and structural diversity of the small molecules was conducted, leading to the selection of 1–2 small molecule compounds from each category. This process ultimately resulted in the identification of 41 small molecule compounds that exhibited high affinity, appropriate molecular weights, and diverse structures. Subsequently, the antiproliferative effects of these candidates on gastric cancer HGC-27 cells were evaluated. Among them, 21 compounds exhibited a significant inhibitory effect on cell proliferation (p < 0.05). Further validation via microscale thermophoresis (MST) experiments indicated that FIIN-2 exhibits the strongest binding affinity with the BAG2-CHIP complex, making it a promising lead inhibitor for subsequent functional studies.

### 2.21 MST assays

MST experiments were conducted using the Monolith NT.115 system (NanoTemper Technologies GmbH, Germany) to quantify the interaction between CHIP and FIIN-2. FIIN-2 is an irreversible pan-FGFR inhibitor, and its IC50 values for FGFR1/2/3/4 are 3.09 nM, 4.3 nM, 27 nM and 45.3 nM, respectively. CHIP human recombinant was purchased from OriGene (cat. TP300310, China). The CHIP solutions were prepared in 10 mM PBS (pH 7.4), and FIIN-2 solutions were prepared in 10 mM PBS (5% v/v DMSO, pH 7.4), respectively. The concentration of CHIP was 50 nM, FIIN-2 was titrated from 3.05 ×10^9^ to 1.0 × 10^4^ M, and the mixed solution of CHIP and FIIN-2 contained 0.05% v/v Tween 20. The samples were added to monolithic capillaries (MO L011; NanoTemper Technologies) and subsequently subjected to MST analysis. The dissociation constant was determined by fitting the curve to a single-site model.

### 2.22 Organoid studies

Take approximately 1 cm³ of fresh gastric cancer tissue, rinse with sterile PBS containing dual antibodies, then cut into pieces of approximately 1 mm³ in size. After washing, digest the tumor tissue with type II collagenase at 37°C for 60 minutes. Filter the cells and centrifuge at 1000 rpm, then wash the cells three times with PBS. Then, resuspend the cells in a 1:1 mixture of DMEM/F12 medium and 40 μL of matrix gel, and place them in a 24-well plate. Incubate the plate at 37°C for 15 minutes until the matrix gel solidifies. Add CultTM organoid culture medium containing 10 ng/mL FGF-2 and 100 μg/mL heparin for cultivation.

### 2.23 Statistical analysis

Statistical analyses were performed using SPSS version 26.0 and GraphPad Prism version 9.0. All data were analyzed for normality using the KO Kolmogorov–Smirnov or Shapiro–Wilk normality tests. In terms of data with normality, a two-sided Student’s t-test was used for two groups and one-way ANOVA was performed for multiple groups, followed by the *post hoc* LSD method (homogeneity of variance) or Tamhane method (heterogeneity of variance). Nonparametric tests were applied for non-normally distributed values. Kaplan–Meier analysis and log-rank tests were performed for survival data. Statistical significance was set at p < 0.05. In the result presentation, “*p<0.05” was used respectively to indicate differences, and “**p<0.01” was used for the table. The difference is significant, and “***p<0.001” indicates a very significant difference.

## 3 Results

### 3.1 BAG2 expression is upregulated in gastric cancer and correlates with poor patient prognosis

**Figure 1:**
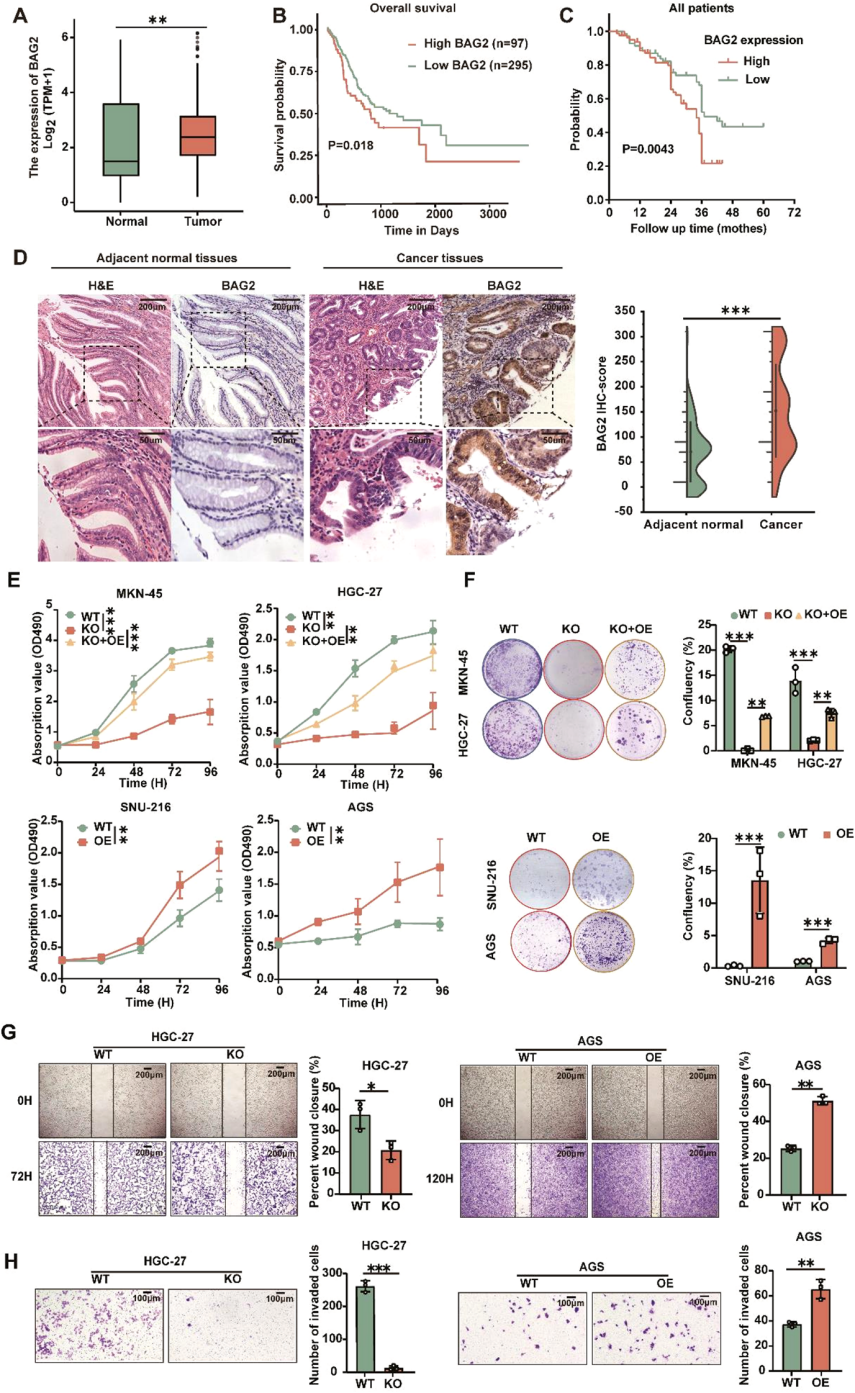
Overexpressed BAG2 correlates with poor clinical outcomes in GC, promoting GC cell line proliferation, invasion, and migration. **(A)** BAG2 transcription level in TCGA database (https://portal.gdc.cancer.gov). **(B)** Prognostic analysis of BAG2 in GC revealed a correlation between high BAG2 expression and poor prognosis, as reported in TCGA. **(C)** Kaplan–Meier survival analysis was conducted to compare the survival outcomes of patients categorized into BAG2-low (n = 75) and BAG2-high (n = 77) groups. **(D)** Representative images of H&E staining and IHC analysis were used to visualize BAG2 expression, and the resulting IHC scores were statistically analyzed. The p values were determined using a two-sided nonparametric test, based on a sample size of 152 independent biological samples. **(E)** The growth curves of several GC cell lines were plotted. Data are presented as means ± SEM. The p values were determined using a two-sided nonparametric test and one-way ANOVA (n = 6 independent biological samples). **(F)** Colony formation assays were conducted in several GC cell lines, and the resulting colonies were counted and statistically analyzed. Data are presented as means ± SEM. The p values were determined via one-way ANOVA (n = 3 independent biological samples). **(G, H)** The effects of BAG2 on GC cell invasion and migration were assessed. Data are presented as means ± SEM. The p values were determined via one-way ANOVA (n = 3 independent biological samples). **p<0.01, ***p<0.001.

To investigate the role of BAG2 in human GC, we performed data mining analysis in publicly available datasets using TCGA. In non-matched samples, we found that BAG2 expression in GC was significantly higher than that in the normal gastric mucosa (**Figure 1A**). We also analyzed the prognosis of BAG2 using RNA samples from 392 cases of GC in TCGA. The results showed that high BAG2 expression in GC was associated with poor prognosis (**Figure 1B**). To further verify whether BAG2 is upregulated in GC, we performed immunohistochemical (IHC) analysis of tissue microarrays (TMAs), including 152 paired GC tumors and adjacent normal tissues, and found that BAG2 was highly expressed in GC samples and that its expression was positively correlated with poor prognosis in GC (**Figures 1C, D**).

### 3.2 BAG2 promotes the proliferation, invasion, and migration of gastric cancer cells *in vitro*

BAG2 expression has been observed to be elevated in gastric cancer tissues and correlates with the process of gastric carcinogenesis. Therefore, we hypothesized that BAG2 plays a key role as a driver in the proliferation, invasion, and migration of gastric cancer cells. To verify this hypothesis, we further investigated the expression levels of BAG2 mRNA and protein in the normal gastric mucosa cell line GES and various gastric cancer cell lines, including MKN-45, HGC-27, SNU-216, AGS, and NCI-N87, using quantitative real-time polymerase chain reaction (qRT-PCR) and immunoblotting (**Supplementary Figures 1A, B**). In addition, we employed CRISPR/Cas9 technology to deplete BAG2 in MKN-45 and HGC-27 cells, followed by the restoration of BAG2 expression. Furthermore, BAG2 was overexpressed in SNU-216 and AGS cells using BAG2-specific lv-siRNA (**Supplementary Figures 1C–F**). MTT experiments demonstrated that the proliferation of BAG2 knockdown (KO) cells was significantly inhibited compared to wild-type (WT) cells, whereas the restoration of cell proliferation was observed in BAG2 overexpressing KO cells (KO + OE group) (**Figure 1E**). These findings were further confirmed by clone formation experiments (**Figure 1F**). Furthermore, MTT and clone formation assays were performed on cell lines overexpressing BAG2, revealing that BAG2 overexpressing promoted GC progression in AGS and SNU-216 cells (**Figures 1E, F**). It is well established that metastasis and invasion of tumor cells lead to the formation of secondary tumors in other tissues and organs, which is an important contributor to cancer-related mortality and morbidity. In light of this, we investigated the effect of BAG2 on the invasion and migration of HGC-27 and AGS gastric cancer cells. The findings of this study demonstrate that the inhibition of BAG2 significantly suppresses the invasion and migration of gastric cancer cells in Transwell and scratch assays conducted on matrix-coated surfaces. Conversely, BAG2 over-expression elicited the opposite effect (**Figures 1G, H**). In summary, the findings underscore the pivotal role of BAG2 in the genesis and progression of gastric cancer.

### 3.3 Regulation of apoptosis in gastric cancer cells by BAG2 *in vivo* and *in vitro*

**Figure 2:**
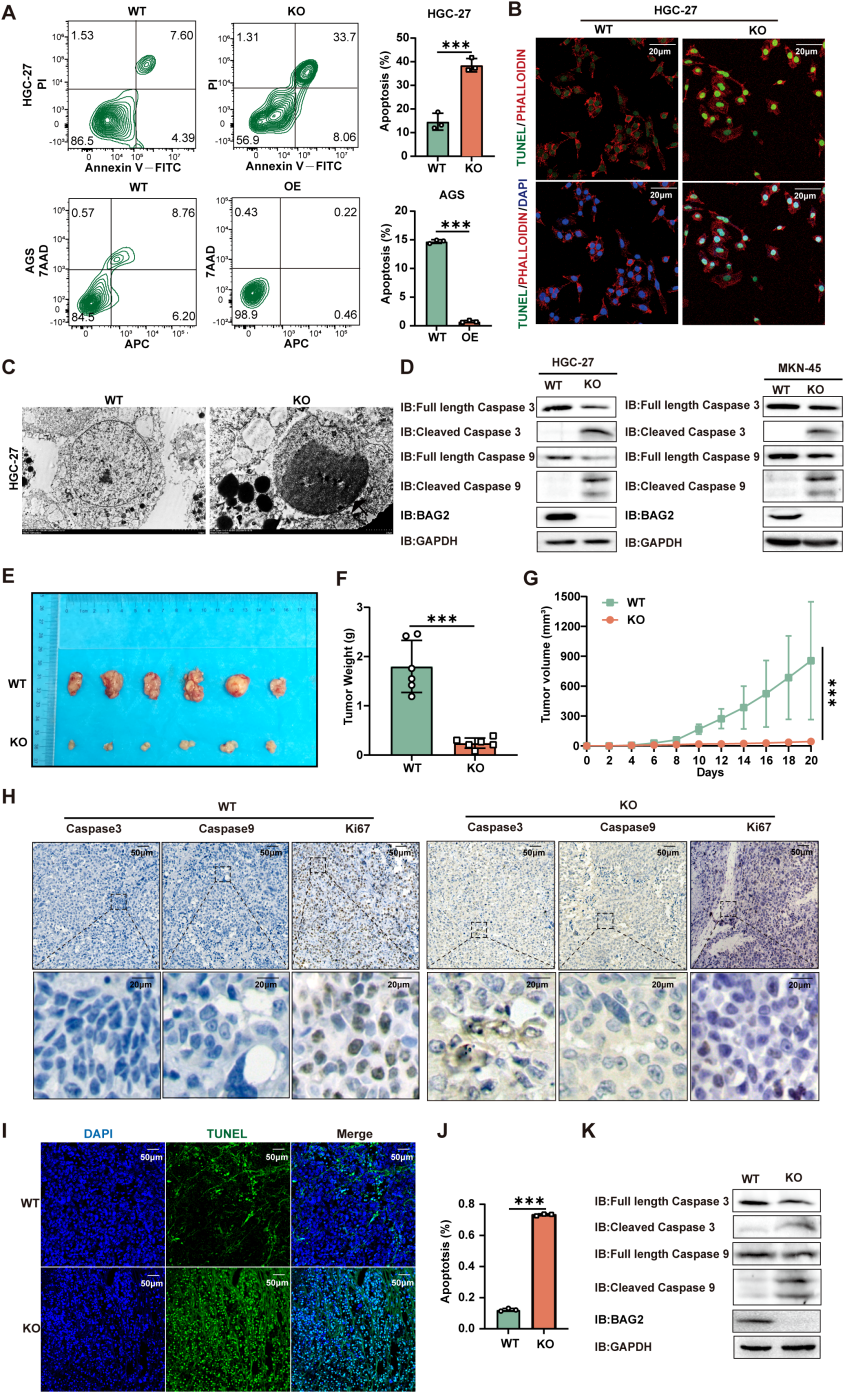
BAG2 KO promotes apoptosis in GC cells. **(A)** Annexin V-FITC/propidium iodide (PI) and Annexin-V APC/7AAD staining tested HGC-27 and AGS cell apoptosis using flow cytometry. Data are presented as means ± SEM. The p values were determined via one-way ANOVA (n = 3 independent biological samples). **(B)** TUNEL staining showed apoptotic changes in HGC-27 GC cells. Scale bar: 100 nm. **(C)** Transmission electron microscopy was used to observe the apoptosome morphology in HGC-27 cells. Scale bar: 50 nm. **(D)** Immunoblotting analysis was performed to examine the protein expression levels of apoptosis factors (including caspase-3 and caspase-9) in BAG2-KO and wild-type cell lines. **(E)** Representative images of xenograft mice carrying WT or BAG2-KO MKN-45 cell xenografts. Mice were sacrificed when tumors reached 100 mm3 in size; data are presented as means ± SEM. The p values were determined through a two-sided nonparametric test (n = 6 independent mice per group). **(F, G)** Tumor weight and growth curve of xenografts of MKN-45 cells with WT or BAG2-KO in mice are presented as means ± SEM. The p-value was determined via two-tailed nonparametric testing and one-way ANOVA (n = 6 independent biological samples). **(H)** Representative intratumor IHC images of caspase-3, caspase-9, and Ki-67, along with quantification of Ki-67-positive cells, are presented for mice xenograft tumors treated with BAG2 KO. **(I, J)** TUNEL staining was used to detect the effect of BAG2 KO on apoptosis in tumor tissues (n=3). Scale bar: 100 nm. **(K)** Representative western blot images of caspase-3, caspase-9, and GAPDH protein expressions in tumor tissues in BAG2-KO and control groups. ***p<0.001.

BAG2, a constituent of the BAG protein family, plays a regulatory role in the process of apoptosis. Prior studies have centered on its role in cell growth and its anti-apoptotic properties. The present study aims to delve into the specific function of BAG2 in apoptosis. To this end, we employed a KO assay to deplete BAG2 expression in gastric cancer cells. The results demonstrated that the proliferation of BAG2-KO cells was significantly inhibited, suggesting the potential role of BAG2 in regulating cell growth. Annexin V-FITC/PI apoptosis assay was performed to analyze the effect of BAG2-KO on apoptosis. The results demonstrated a notable increase in apoptotic cells in the BAG2-KO group compared to the WT group (**Figure 2A**). These results suggest that BAG2 may have anti-apoptotic effects and that its KO may trigger an increase in apoptosis.

Subsequent assessment of apoptosis via TUNEL assay revealed a greater number of TUNEL-positive cells in the BAG2-KO group, thereby confirming the pro-apoptotic effect of BAG2 KO (**Figure 2B**). Electron microscopy revealed augmented apoptotic body formation in BAG2 KO cells (**Figure 2C**). To further explore the underlying molecular mechanisms, the expression levels of apoptosis-related factors BAX, BCL-2, caspase-3, and caspase-9 were examined by western blot. The results demonstrated that BAG2 KO significantly increased the expression of caspase-3 and caspase-9 in MKN-45 and HGC-27 cells (**Figure 2D**). Conversely, no substantial alterations in BAX or BCL-2 expression were detected in the BAG2 KO cells (**Supplementary Figure 2A**). In summary, our study demonstrated that BAG2 expression is closely related to apoptosis. The knockdown of BAG2 resulted in an increase in the number of apoptotic cells, the formation of apoptosome, and the up-regulation of caspase-3 and -9 levels, thereby supporting the hypothesis that BAG2 exerts an inhibitory effect on apoptosis.

Furthermore, a nude mouse transplantation tumor model with MKN-45 cells was established to further explore the effect of BAG2 on tumor growth *in vivo*. Consistent with the results of the *in vitro* experiments, BAG2 KO significantly reduced tumor volume and slowed down tumor growth (**Figures 2E–G**). To elucidate the potential mechanisms involved, the expression levels of apoptotic factors (e.g., HE, caspase-3, and caspase-9) and cell proliferation index (Ki67) were detected by immunohistochemical staining and protein blotting in nude mice. The results demonstrated that BAG2 KO significantly augmented the protein expression of caspase-3 and caspase-9 in tumor tissues compared with controls (**Figure 2H**; **Supplementary Figure 2B**). These findings collectively indicate that BAG2 KO promotes apoptosis in tumor tissues by activating the caspase pathway. To further validate these findings, a TUNEL assay was performed to evaluate the apoptosis rate in the xenograft model tissues. The results demonstrated a significant increase in the number of TUNEL-positive cells (green fluorescence) in the KO group compared to the WT group (**Figures 2I, J**). Furthermore, tissue-level protein blotting experiments verified that downregulation of BAG2 resulted in increased expression levels of cleaved caspase-3 and cleaved caspase-9 in tumor tissues (**Figure 2K**). Collectively, these findings further substantiate that BAG2 knockdown promotes apoptosis in gastric cancer cells, leading to a substantial reduction in tumor volume and growth rate *in vitro* and *in vivo*.

### 3.4 Newly identified BAG2-CHIP-HSP70-Apaf1/Cytc axis involved in apoptosis

**Figure 3:**
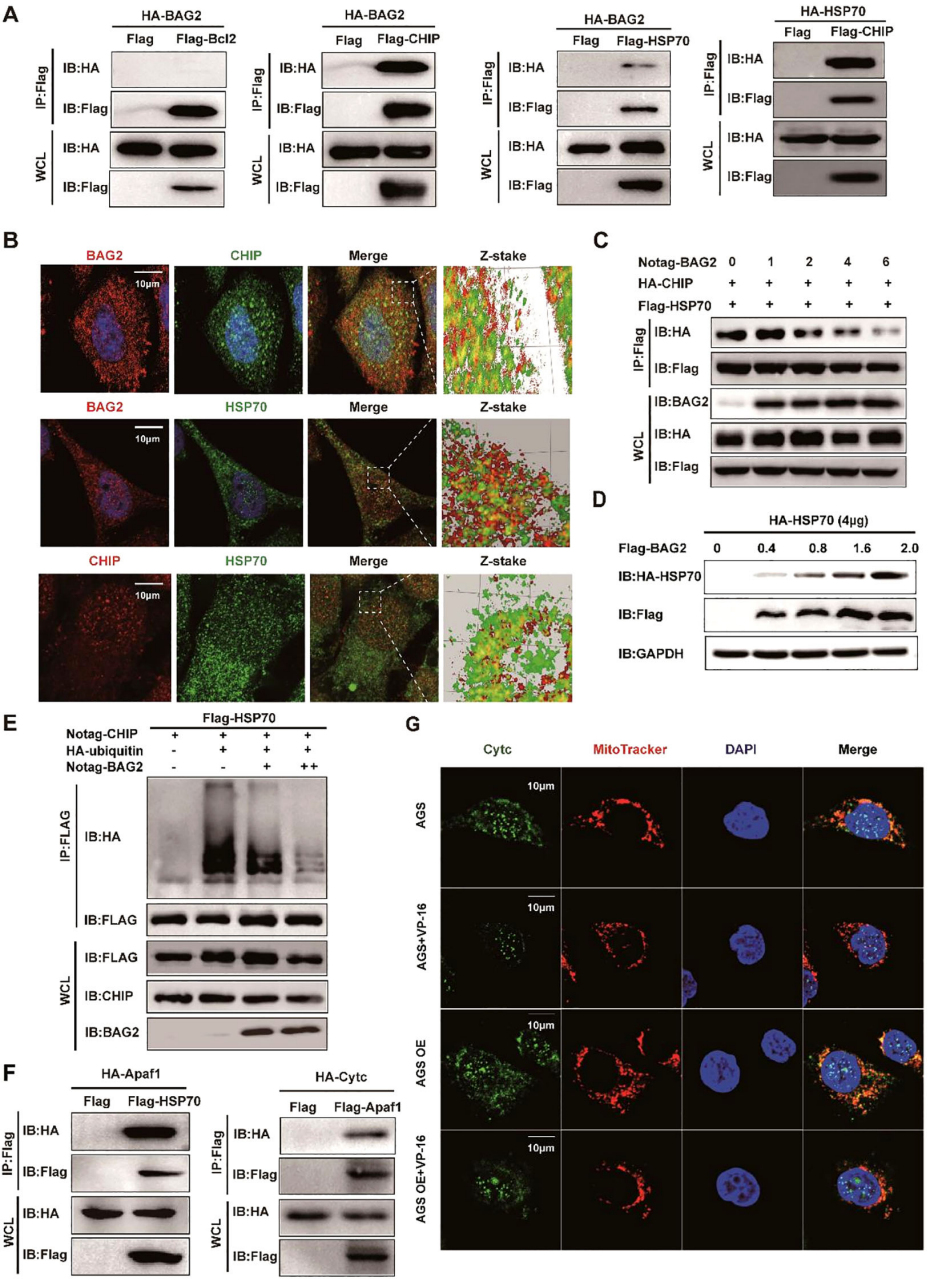
BAG2 regulates GC cell apoptosis through the CHIP-HSP70-Apaf1/Cyt-c axis. **(A)** Co-immunoprecipitation assays of Flag/HA-tagged BCL2, BAG2, CHIP, or HSP70 co-expressed in HEK293T cells. IP, immunoprecipitation; WCL, whole-cell lysates. **(B)** Immunofluorescence colocalization of BAG2 and CHIP/HSP70 in AGS cells. Cells were immunostained with anti-BAG2 antibody (red), anti-CHIP antibody (red or green), anti-HSP70 antibody (green) and DAPI (blue). **(C)** Co-immunoprecipitation of HA-tagged CHIP and FLAG-tagged HSP70 was performed in HEK-293 cells transfected with No-tagged BAG2. **(D)** HEK-293T cells were transiently transfected with plasmids encoding HA-tagged HSP70, along with the indicated amounts of a plasmid encoding Flag-BAG2 24 h after transfection. Cell lysates were analyzed via western blotting with the indicated antibodies. **(E)** HEK-293T cells were transiently transfected with plasmids encoding Flag-tagged HSP70 along with plasmids encoding HA-tagged wild ubiquitin or indicated mutant ubiquitin. Sixteen hours after transfection, cells were treated with MG132 for 8 h (10 μM). Cell lysates were analyzed via immunoprecipitation with anti-Flag and western immunoblotting with indicated antibodies. **(F)** Co-immunoprecipitation assays of Flag-tagged HSP70 and HA-tagged Apaf1 co-expressed in HEK-293T cells and co-immunoprecipitation assays of Flag-tagged Apaf1 and HA-tagged Cytc co-expressed in HEK-293T cells. IP, immunoprecipitation; WCL, whole-cell lysates. **(G)** Immunofluorescence colocalization of DAPI, Cytc, and mitochondria in AGS cells. Cells were immunostained with anti-Cytc antibody (green), MitoTracker^®^ Red CMXRos (red), and DAPI (blue).

In light of these findings, a hypothesis was formulated proposing the involvement of BAG2 in the process of gastric carcinogenesis. To elucidate the subpopulation of BAG2-binding proteins and to identify undiscovered downstream axes of BAG2, we co-expressed Flag- or ha-tagged BCL2, BAG2, CHIP, and/or HSP70 in HEK-293T cells. The immunoprecipitation assay revealed the formation of the BAG2-CHIP-HSP70 complex (**Figure 3A**). Furthermore, immunofluorescence co-localization analysis revealed the presence of BAG-2/Hsp70/CHIP ternary complexes in gastric cancer cells (**Figure 3B**). Furthermore, immunoprecipitation experiments substantiated that BAG2 formed complexes with CHIP and HSP70 (**Supplementary Figure 3**). *In vitro* experiments demonstrated that BAG2 interfered with the interaction of CHIP with HSP70 in a concentration-dependent manner (**Figure 3C**). Furthermore, the data demonstrated that BAG2 increased the protein level of HSP70 in a dose-dependent manner and inhibited the ubiquitinated degradation of HSP70 (**Figures 3D, E**). Subsequently, we sought to determine whether the function of BAG2 in gastric cancer progression is mediated through the inhibition of ubiquitination and degradation of HSP70. *In vivo*, ubiquitination assays demonstrated that BAG2 overexpression suppressed HSP70 degradation. It has been demonstrated that HSP70 exerts a pivotal regulatory function in apoptosis triggered by diverse stimulants. Moreover, HSP70 has been demonstrated to inhibit apoptosis by directly binding to Apaf-1 and impeding the formation of the apoptotic complex. To demonstrate the direct link between HSP70 and Apaf-1, we co-expressed Flag-tagged HSP70 and HA-tagged Apaf-1 in HEK-293T cells and found that Apaf-1 interacted with endogenous HSP70 by immunoprecipitation assay (**Figure 3F**). Additionally, the interaction between Apaf1 and Cytc was verified using the same method (**Figure 3F**). The findings of this study demonstrate that the presence of VP-16 results in the release of Cytc from the mitochondria, consequently inducing apoptosis. Conversely, no indications of apoptosis were observed in cells overexpressing BAG2, suggesting its potential protective role (**Figure 3G**). Consequently, these results collectively suggest that the BAG2/CHIP axis promotes apoptosis in gastric cancer cells by impeding apoptosome assembly.

### 3.5 BAG2 regulates the proliferation and apoptosis of gastric cancer cells through HSP70

**Figure 4:**
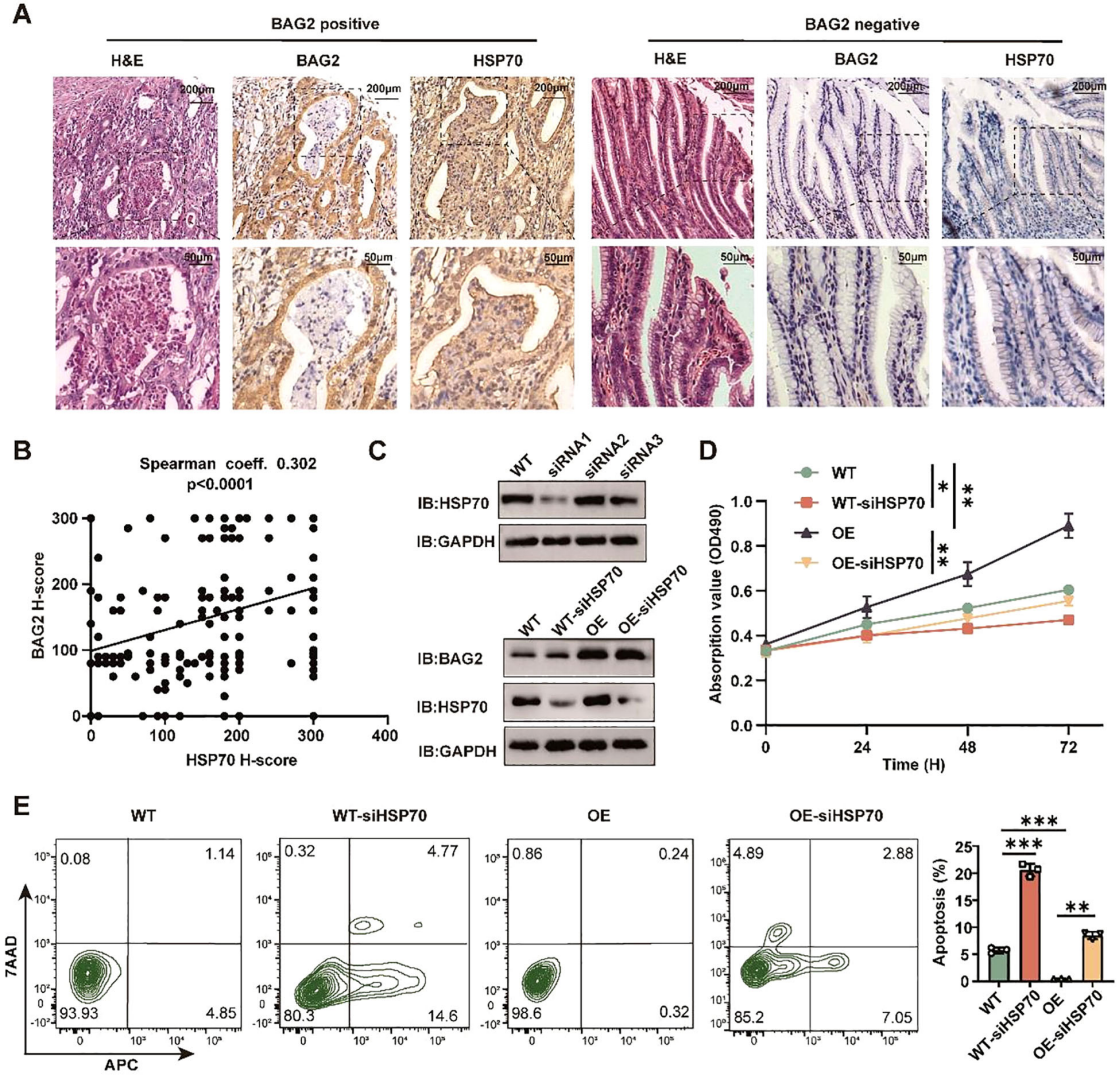
BAG2 regulates the proliferation and apoptosis of GC cells dependent on HSP70. **(A)** Representative H&E and IHC staining of BAG2 and HSP70 in GC TMAs. **(B)** Scatter plots of BAG2 versus HSP70 H scores in human gastric TMAs. P values were determined using a two-sided Spearman’s rank correlation test (n = 185 independent biological samples). **(C)** Western blot was performed to validate the knockdown efficiency of three specific siRNAs against HSP70 in GC cells (n=3). **(D)** An MTT assay was conducted to validate the effect of HSP70 knockdown on the proliferation of GC HGC-27 cells. **(E)** Flow cytometry was performed to validate the effect of HSP70 knockdown on the apoptosis of HGC-27 cells(n=3). *p<0.05, **p<0.01, ***p<0.001.

In order to investigate the up-regulation of BAG2 expression in gastric cancer tissues and its correlation with HSP70 amplification, hematoxylin-eosin (HE) staining and immunohistochemical staining of 185 pairs of tissue microarrays (TMAs) composed of gastric cancer and paracancerous normal tissues were performed. The results demonstrated a significant moderate correlation between BAG2 and HSP70 expression (Spearman’s coefficient = 0.302, **Figures 4A, B**).

To ascertain the dependence of gastric cancer cell proliferation and apoptosis on BAG2 and HSP70, three distinct siRNAs targeting WT and overexpressing BAG2 in gastric cancer cells were utilized. Subsequent experiments were executed using the most efficient KO cellular (**Figures 4C, D**). The results of MTT and flow cytometry demonstrated that in WT and gastric cancer cells overexpressing BAG2, HSP70 KO significantly curtailed proliferation and induced apoptosis (**Figure 4E**). In summary, the present study has yielded evidence that lends support to the hypothesis of BAG2 upregulation in gastric cancer tissues, as well as its moderate correlation with HSP70 expression. Furthermore, we found that the regulatory effects of BAG2 on the proliferation and apoptosis of gastric cancer cells were dependent on HSP70.

### 3.6 BAG2-CHIP interaction patterns using virtual screening

**Figure 5:**
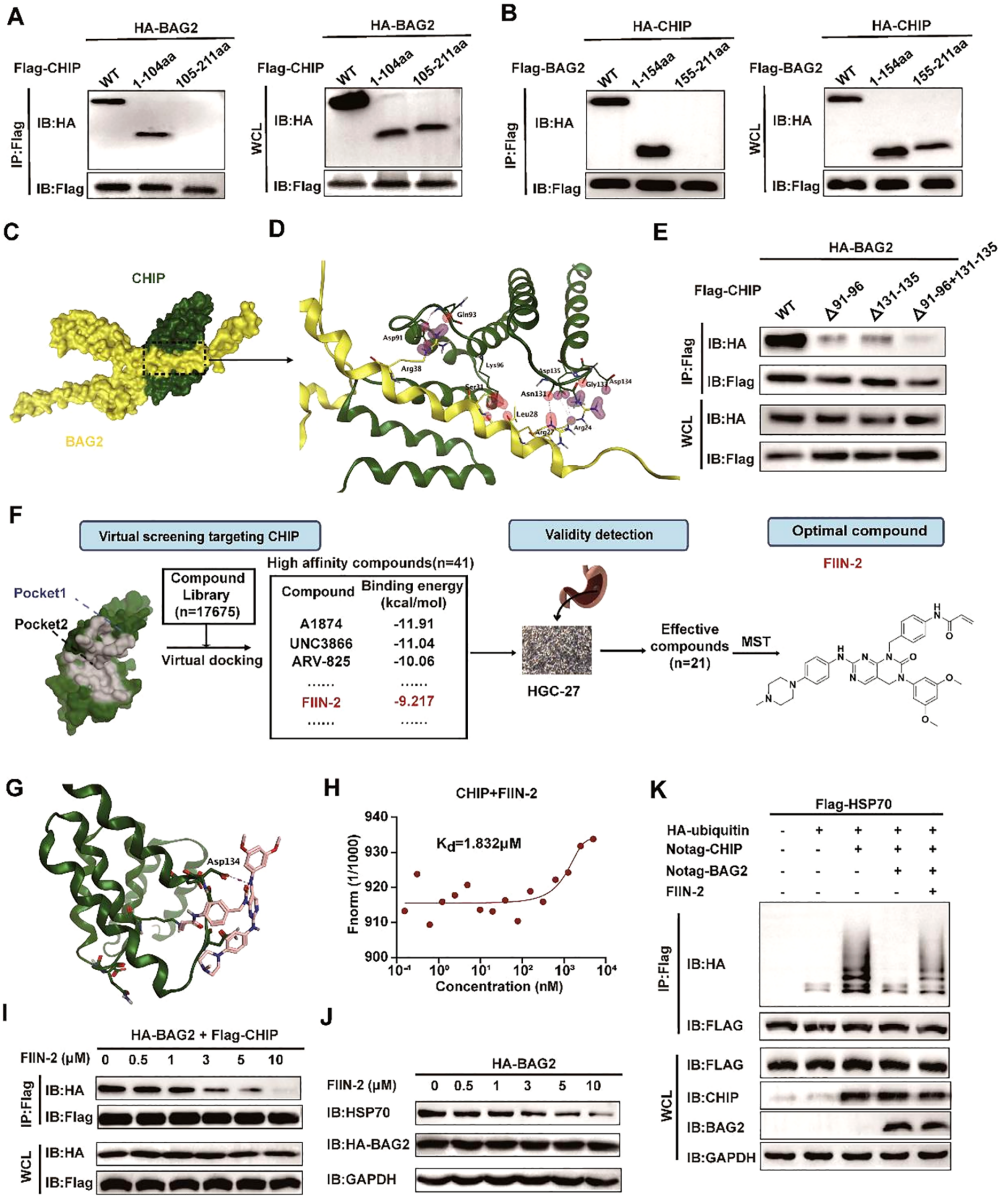
FIIN-2 blocks the BAG2-CHIP interaction. **(A)** Interactions between CHIP and different BAG2 deletion mutants analyzed via co-immunoprecipitation assays. WT, wild type; IP, immunoprecipitation; WCL, whole-cell-lysates. **(B)** Interactions between BAG2 and different CHIP deletion mutants were examined using co-immunoprecipitation assays. **(C)** The bound conformation of BAG2 and CHIP as predicted by the Cluspro algorithm. BAG2 is displayed in yellow, and CHIP is displayed in green. **(D)** This schematic diagram shows the amino acids that interact between BAG2 and CHIP. On the binding surface, the CHIP residue bonds are highlighted in green, while those of BAG2 are in yellow. **(E)** Interactions between BAG2 and different CHIP deletion mutants containing residues on the binding surface of the mode were analyzed via co-immunoprecipitation assays. **(F)** Flow diagram of BAG2-CHIP complex inhibitor screening. **(G)** Computational modeling showcases the interactions between FIIN-2 and CHIP. CHIP is displayed in green, and FIIN-2 is displayed in pink. **(H)** Microscale thermophoresis (MST) was utilized to ascertain the kinetic constant (Kd) for the interaction between FIIN-2 and CHIP. **(I)** Co-immunoprecipitation assays of the BAG2-CHIP interaction in cells treated with FIIN-2 at the indicated concentrations in HGC-27. IP, immunoprecipitation; WCL, whole-cell lysates. **(J)** Western blotting was conducted to assess HSP70 expression levels in cells post-treatment with different FIIN-2 concentrations in HGC-27. **(K)** An *in vitro* ubiquitination assay was performed to determine the impact of FIIN-2 (C = 10 μM) on HSP70 ubiquitination, using specified recombinant proteins in HGC-27.

Subsequent to confirming that BAG2 knockdown promotes apoptosis through activation of the BAG2-CHIP-HSP70-Apaf1/Cytc axis, the hypothesis was formulated that pharmacological inhibitors targeting the BAG2-CHIP complex could potentially inhibit the proliferation of gastric cancer cells while promoting apoptosis. To develop functional inhibitors of BAG2-CHIP binding, we sought to identify BAG2 peptides capable of binding to CHIP. Structural domain truncation experiments and immunoprecipitation demonstrated that amino acid residues 1–104 of BAG2 are necessary for binding CHIP (**Figure 5A**). Concurrently, we identified the critical binding site of BAG2 to CHIP involving amino acids 1–154 of CHIP (**Figure 5B**).

Utilizing the computational protein-protein docking algorithm Cluspro, we subsequently predicted the binding conformations of the CHIP and BAG2 structural domains and determined their structures by x-ray analysis. The prediction analysis revealed that Cluspro selected the highest-level binding conformation for the BAG2-CHIP complex, thereby exposing the seven core amino acids on its binding surface (**Figures 5C, D**). To validate this predicted BAG2-CHIP binding mode, we generated protein deletion mutations and amino acid mutations in CHIP, encompassing the seven core amino acids on the contact surface. Immunoprecipitation analysis was then performed between the BAG2 and CHIP mutations, revealing that mutations encapsulating two amino acid fragments (91–96 aa and 131–135 aa) effectively disrupted the binding link between CHIP and BAG2 (**Figure 5E**). This finding serves to validate our proposed BAG-CHIP complex model, in which the 91–96 amino acid fragments and the 131–135 amino acid fragments are identified as the core amino acids on the contact surface.

### 3.7 FIIN-2 is an inhibitor of the BAG2-CHIP complex

Following the identification of the significance of residues 91–96 aa and 131–135 aa of the CHIP protein, a small-molecule inhibitor was successfully identified. This inhibitor, designated as FIIN-2, specifically targets these critical residues through a combination of virtual screening and experimental validation. Structural analyses of CHIP revealed that its binding site is located in the binding pocket of the two inhibitors (**Figure 5F**; **Supplementary Figure 4A**). This finding provided a structural basis for the subsequent screening of potential inhibitors.

In order to identify potential inhibitors of the BAG2-CHIP interaction, a virtual docking screening was performed on a library of 17,675 bioactive small molecules (**Supplementary Figures 4B, C**). The initial selection criteria included binding affinity, molecular weight (MW < 500 Da), and structural diversity. After excluding repetitive and aliphatic chain compounds, we prioritized 1–2 representative candidates from each scaffold cluster, yielding 41 high-affinity compounds with favorable drug-like properties. Subsequently, the anti-proliferative effects of these candidates on gastric cancer HGC-27 cells were evaluated using the MTT assay. Among them, 21 compounds significantly suppressed cell proliferation (*P* < 0.05). Further validation via MST assays revealed that FIIN-2 exhibited the strongest binding affinity to the BAG2-CHIP complex, with a Kd value of 1.832 μM, establishing it as a lead inhibitor for subsequent functional studies (**Figures 5G, H**). This finding suggests that FIIN-2 possesses a favorable binding affinity.

Moreover, FIIN-2 demonstrated a substantial inhibitory effect on the interaction between BAG2 and CHIP in *in vitro* experiments. As the concentration of FIIN-2 increased, the co-precipitation signal between BAG2 and CHIP was significantly reduced, suggesting that FIIN-2 effectively blocked this critical interaction (**Figure 5I**). Furthermore, FIIN-2 significantly reduced the inhibitory effect of BAG2 on HSP70 ubiquitination (**Figures 5J, K**), suggesting that FIIN-2 exerts its influence not only on the binding of BAG2 and CHIP but also on the homeostasis of intracellular proteins through other mechanisms.

The discovery of FIIN-2 is significant and innovative in that it provides a small molecule tool to specifically block the interaction between BAG2 and CHIP. Furthermore, it demonstrates the potential application of FIIN-2 in anticancer therapy. These findings establish a substantial foundation for the further development of FIIN-2-based anticancer pharmaceuticals.

### 3.8 FIIN-2 has a significant inhibitory effect on gastric cancer by inducing apoptosis

**Figure 6:**
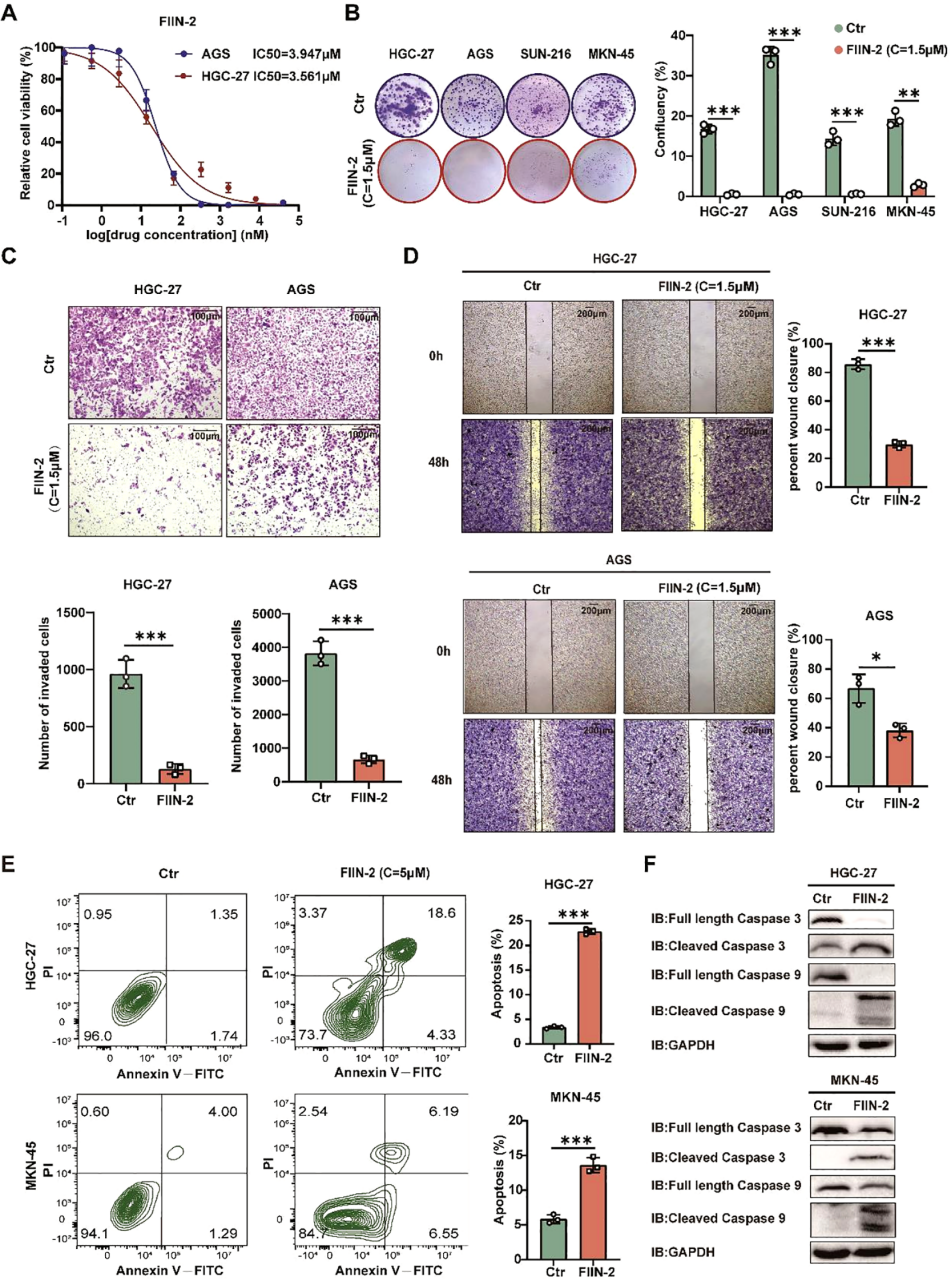
FIIN-2 is efficient in GC treatment *in vitro*. **(A)** Sensitivity of HGC-27 and AGS cells to FIIN-2 at various concentrations. Cell proliferation was evaluated after 48h of treatment. The results are expressed as means ± SEM. The p values were determined via one-way ANOVA (n = 4 independent biological samples). **(B-D)** Effect of FIIN-2 treatment on tumor **(B)** proliferation, **(C)** invasion, and **(D)** migration ability were detected via colony formation, Transwell, and wound-healing assays. Student’s t-test was used to examine statistical significance (mean ± SD, n = 3, ****p < 0.0001, ***p < 0.001,**p < 0.01, *p < 0.05). **(E, F)** Effect of FIIN-2 treatment on tumor apoptosis were detected via flow cytometry and western blot analysis (n=3).

In the present study, the inhibitory effect of FIIN-2 on bag2-driven gastric cancer progression was investigated. The study examined the sensitivity to FIIN-2, revealing that increasing the dosage of FIIN-2 led to a substantial reduction in the proliferation of HGC-27 and AGS cells (**Figure 6A**). The capacity of FIIN-2 to impede proliferation was also observed in HGC-27, AGS, SUN-216, and NKN-45 gastric cancer cells (**Figure 6B**). In addition, data obtained from matrix-coated Transwell and wound healing assays clearly illustrated the potent effectiveness of FIIN-2 in limiting the invasive and migratory abilities of HGC-27 and AGS cells, emphasizing its effectiveness against GCs (**Figures 6C, D**). Furthermore, the results of the flow cytometry analysis and western blot assay confirmed that FIIN-2 induced apoptosis in HGC-27 and MKN-45 gastric cancer cells. Among the other notable findings were the increased expression levels of caspase-3 and -9 cleavage fragments, respectively (**Figures 6E, F**). In summary, the findings underscore the promise of FIIN-2 as a promising therapeutic agent for bag2-driven gastric cancer progression.

**Figure 7:**
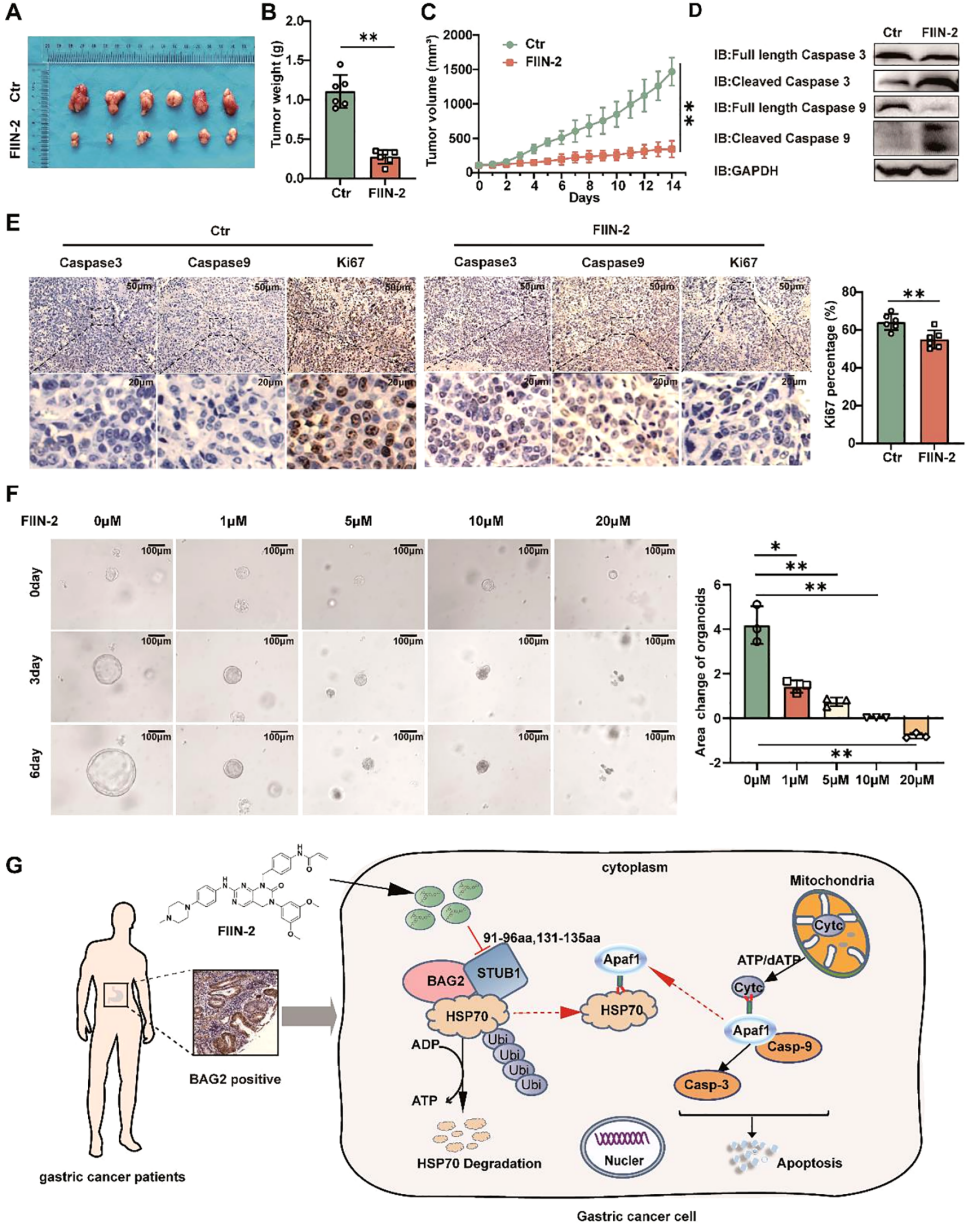
FIIN-2 is efficient in treating GC *in vivo* and in organoids. **(A-C)** MKN45 cells were intratumorally injected in nude mice. The compound was dosed by intraperitoneal injections at a single dose of 20mg/kg/day×21, and DMSO was used as a control group. Shown are **(A)** representative images, **(B)** tumor weights, and **(C)** tumor volumes. Data are the mean ± s.e.m. The p values were determined by one-way ANOVA (n = 6 independent biological samples). **(D)** The effect of drug administration on caspase-3 and caspase-9 expression in tumor tissues was assessed using western blot analysis. **(E)** Representative intratumor IHC images of caspase-3, caspase-9, and Ki-67, along with quantification of Ki-67-positive cells, are presented for mice xenograft tumors treated with FIIN-2. Data represent the mean ± SEM. The p values were determined using one-way ANOVA with n = 6 independent biological samples. **(F)** The effect of FIIN-2 on GC organoids at various concentrations(n=3). **(G)** A schematic diagram of the mechanism by which FIIN-2 obstructs the progression of GC by blocking the BAG2-CHIP complex and regulating apoptosis mediated by HSP70 ubiquitination. *p<0.05 is used to indicate a difference, **p< 0.01 to indicate a significant difference.

The present study investigated the impact of FIIN-2 on the growth of MKN-45 xenografts in mice. The optimal tumor volume for FIIN-2 treatment was determined to be approximately 125 mm3. A significant reduction in tumor growth was observed after two weeks of intraperitoneal injection of a dose of 20 mg/kg per day (**Figures 7A-C**). The results showed a surge in the levels of activated caspase-3 and caspase-9, which are markers of apoptosis induction (**Figure 7D**). A comprehensive examination of IHC staining of HE, Ki-67, caspase-3, and caspase-9 positive cells within the tumor tissues revealed a substantial decrease in cell proliferation and a concomitant increase in apoptosis in the treated group (**Figure 7E**; **Supplementary Figure 5**). This evidence strongly suggests that FIIN-2 stimulates the apoptotic destruction of tumor cells. Furthermore, a human gastric cancer organoid model was established and divided into five groups: a control group and four FIIN-2 treatment groups at concentrations of 1 μM, 2 μM, 5 μM, and 10 μM. The inhibitory effects of different FIIN-2 concentrations on gastric cancer organoids were evaluated. Results showed that, within the 5-10 μM concentration range, FIIN-2 significantly inhibited gastric cancer organoids. (**Figure 7F**). These observations underscore the pivotal role of FIIN-2 in restraining cell proliferation and promoting apoptosis. This study underscores the promise of FIIN-2 as a potential anticancer drug for the treatment of gastric cancer. A substantial body of evidence supports the hypothesis that FIIN-2 has the potential to further develop therapeutic drugs for gastric cancer by inhibiting tumor growth, promoting apoptosis, and inhibiting cell proliferation.

## 4 Discussion

Apoptosis is a multistep and complex process that plays a dual role in cancer. On the one hand, it helps maintain tissue homeostasis and remove damaged or abnormal cells, thus preventing carcinogenesis. On the other hand, cancer cells are often able to evade the apoptotic mechanism, which contributes to tumor growth and proliferation (18–20). BAG2, a molecular chaperone cofactor, plays a substantial role in various cancers, particularly gastric cancer (GC), where it contributes to the anti-apoptotic process (21–24). The present study explores the interaction of BAG2 with CHIP and its impact on HSP70 ubiquitination, Cyt c release, and the apoptotic pathway. Furthermore, the potential function of BAG2 in the assembly of apoptosome and its role in maintaining genomic stability are addressed. A critical direction for developing novel anti-cancer therapeutic strategies for GC patients is understanding and interfering with the apoptotic escape mechanisms of cancer cells.

The present study offers preliminary evidence suggesting that BAG2 levels are elevated in gastric cancer samples compared to their corresponding normal tissue. Higher BAG2 expression levels were found to be associated with a worse prognosis in patients with GC. Furthermore, the knockdown of BAG2 significantly inhibited the proliferation, invasion, and migration of gastric cancer cells. Furthermore, our study identified BAG2 as a key gene that promotes the malignant progression of gastric cancer through the apoptotic pathway. Previous studies have shown that BAG2 interacts with CHIP and participates in the ubiquitin-proteasome system by ubiquitinating misfolded proteins associated with cytoplasmic chaperones. bAG2 inhibits the ubiquitination of CHIP misfolded proteins through an auxiliary chaperone-dependent regulatory mechanism. Our study corroborates these findings by demonstrating that BAG2 interacts with CHIP, thereby impeding the ubiquitinated degradation of HSP70 (**Figure 7G**). This, in turn, results in an increase in the binding of HSP70 to Apaf1 and a concomitant decrease in the release of mitochondrial Cytc. The subsequent inhibition of the apoptosome, which is assembled by Cytc and Apaf1, and the downstream activation of caspase-9/3, ultimately contributes to the observed effects (25). Consequently, the observed inhibition of apoptosis and the concomitant increase in the malignancy of gastric cancer are notable findings.

Apoptosome are intracellular structures that form during apoptosis, playing a crucial role in cell death (26, 27). These vesicles are assembled from activated proteins, such as caspase-9, Apaf-1, and Cytc (28, 29). Their formation is a key step in the apoptotic signaling pathway, responsible for activating the effector cysteine aspartate enzyme, which ultimately leads to cellular death (30). The assembly of these vesicles is a highly regulated process involving multiple regulatory proteins. Abnormalities in the process of apoptotic vesicle assembly have been demonstrated to promote tumor formation, growth, and spread. Research has demonstrated that HSP70 exerts a significant paracrine function in the process of apoptotic vesicle assembly. It has been demonstrated that HSP70 exerts its function by preventing the formation of apoptosome, a process that is facilitated by the inhibition of the decrease in mitochondrial membrane potential and the release of cytochrome C (31). In gastric cancer cells with high expression of BAG2, the process of apoptotic vesicle assembly may be optimized as the stability of HSP70 is enhanced. This observation lends support to the notion that the BAG2/CHIP axis plays a pivotal role in the regulation of apoptotic vesicle assembly and apoptosis. Consequently, the targeting of apoptotic vesicle assembly emerges as a promising therapeutic strategy for cancer treatment, as it induces apoptosis, thereby impeding the proliferation and metastasis of cancer cells by disrupting the assembly of these vesicular structures.

In light of the aforementioned findings, a small-molecule inhibitor, FIIN-2, was identified and characterized. FIIN-2 was initially identified as an effective inhibitor of the fibroblast growth factor receptor (FGFR) family. FIIN-2 has been demonstrated to target FGFR, thereby influencing a variety of processes, including tumorigenesis, the tumor microenvironment, and tumor resistance (32). Furthermore, FIIN-2 has demonstrated moderate efficacy against the epidermal growth factor receptor (EGFR), the proto-oncogene tyrosine protein kinase Src (SRC), and the tyrosine protein kinase Yes (YES) (33). Research has demonstrated that FIIN-2, a specific inhibitor of FGFR, demonstrates notable antitumor efficacy in the treatment of lung adenocarcinoma. This antitumor activity is attributed to the induction of apoptosis, a process that is initiated by mitochondrial damage. The antitumor efficacy of FIIN-2 is associated with its ability to inhibit FGFR and its downstream signaling pathways, including the PI3K/AKT pathway. Furthermore, it was determined that FIIN-2 exerts its regulatory effects on Bcl-2 and Bax levels through a dose- and time-dependent manner, consequently activating the apoptosis-related protein Caspase-3 and inducing apoptosis in lung adenocarcinoma cells (34). Our study demonstrated that this small molecule can effectively interfere with the interaction between BAG2 and CHIP, thereby reducing the stability of HSP70 and altering the cellular processes in which it participates. This small-molecule drug is hypothesized to exert its anticancer effects through a dual mechanism. First, it is predicted to interfere with the BAG2-CHIP axis to restore normal HSP70 ubiquitination and promote apoptosis. Second, it may inhibit FGF-mediated signaling pathways to block cancer cell proliferation and metastasis. Furthermore, given the established correlation between abnormal FGFR activity and ribosomal biosynthesis as well as chromosomal aberrations (35, 36), it can be hypothesized that pharmacological inhibition of this receptor may have a significant impact on cancer cell nucleosome dynamics and genomic stability. Consequently, the effects of this small-molecule drug extend beyond traditional anti-apoptotic mechanisms, offering a more comprehensive strategy for cancer therapy by interfering with nucleosome assembly and chromosomal stability. Further investigation into the interplay between BAG2 and FGFR signaling in gastric cancer, along with the use of FIIN-2 or the development of more optimized dual-target/selective BAG2 inhibitors, could offer new insights and therapeutic opportunities for targeting apoptosis in gastric cancer cells.

Despite the initial indications of FIIN-2’s effectiveness in impeding BAG2-CHIP-mediated gastric cancer proliferation in both *in vitro* and *in vivo* models, it is acknowledged that the present study is not without its limitations. Firstly, the FIIN-2 inhibitor may pose potential off-target effects risks. It is imperative that future studies employ more comprehensive analyses and methods to systematically identify its off-target sites, assess its potential impact on non-targeted signaling pathways, and provide critical evidence for subsequent structural optimization to enhance target specificity. Secondly, the *in vivo* models utilized in this study predominantly evaluated short-term treatment effects, lacking assessments of long-term efficacy, potential mechanisms of resistance, and chronic toxicity. Long-term *in vivo* data for FIIN-2 or its analogues are relatively scarce. In order to comprehensively assess the therapeutic potential and clinical translational feasibility of FIIN-2, it is imperative that future work prioritize validation through longer-term *in vivo* experiments. Furthermore, continuous monitoring of the dynamic changes in tumors and exploration of potential acquired resistance mechanisms are imperative. A comprehensive evaluation of the potential toxicity of long-term administration on major organs (e.g., the liver, kidneys, and hematopoietic system) and its impact on overall health indicators, including body weight and behavioral changes, is also imperative.

In summary, our study provides a detailed analysis of the complex roles of BAG2 in gastric cancer, revealing its multiple functions in anti-apoptosis, nucleosome assembly, and maintenance of genome stability. By impeding the interaction of BAG2 with CHIP, it was found that the normal apoptotic pathway was restored and that this interaction may trigger multiple cellular effects in cancer cells by affecting nucleosome assembly. Future studies will further validate the clinical potential of this small molecule drug and explore its application value in cancer therapy from multiple perspectives.

## Data Availability

The original contributions presented in the study are included in the article/**Supplementary Material**. Further inquiries can be directed to the corresponding authors.
