# Supplementary material for: Targeting the BAG2/CHIP axis promotes gastric cancer apoptosis by blocking apoptosome assembly
Source: Front Immunol. 2025 Jul 18;16:1578416. doi: 10.3389/fimmu.2025.1578416 (PMC12313710; doi:10.3389/fimmu.2025.1578416)
Supplement: Supplementary file 1 [file DataSheet1.docx]

Supplementary Material

# Supplementary Figures


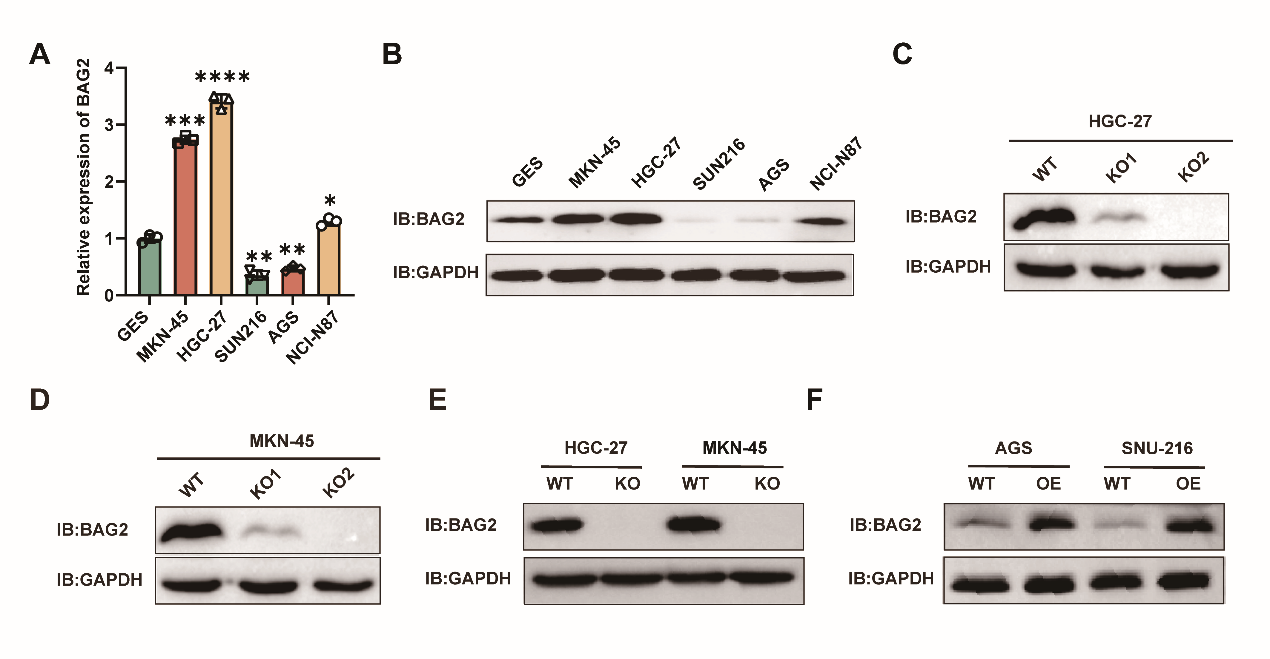


**Supplementary Figure 1.** Establishment of BAG2-KO and BAG2-OE GC cell lines. **(A, B)** BAG2 mRNA and protein expression levels were evaluated in a normal gastric mucosa cell line, GES, as well as several GC cell lines (MKN-45, HGC-27, SNU-216, AGS, NCI-N87). **(C-F)** The interference efficiency of BAG2-specific sgRNA and BAG2-specific LV-sgRNA was assessed.


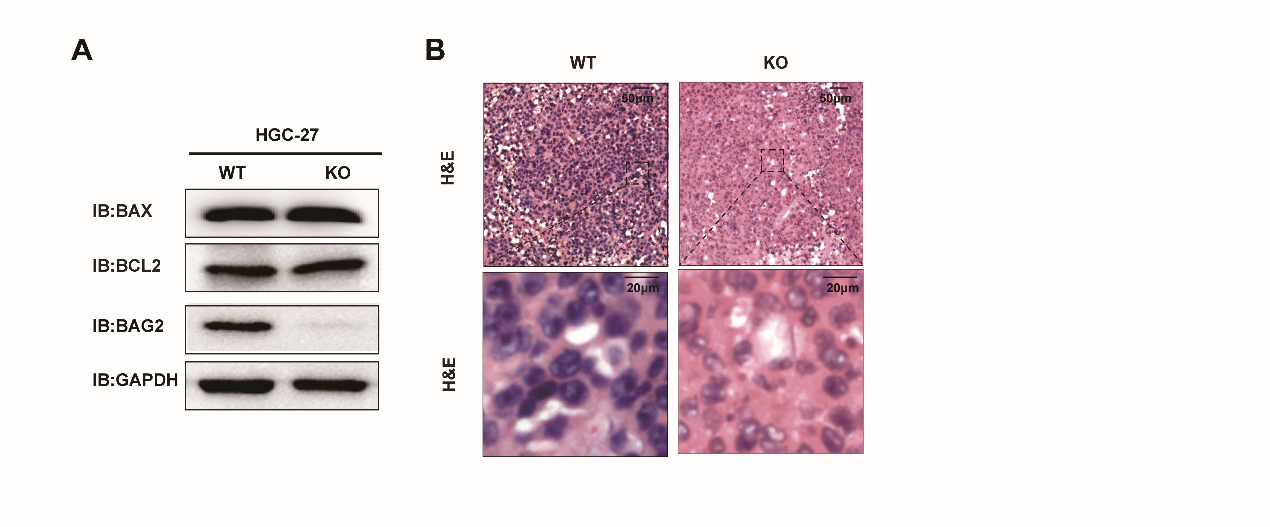


**Supplementary Figure 2.** BAG2 KO has no effect on the expression levels of Bcl-2 and BAX. **(A)** Immunoblots analysis was performed to examine the protein expression levels of apoptotic apoptosis factors (including BAX and BCL-2) in the BAG2 KO and WT cell lines. **(B)** Representative images of hematoxylin–eosin (H&E) in mice xenograft tumors treated with BAG2 KO.


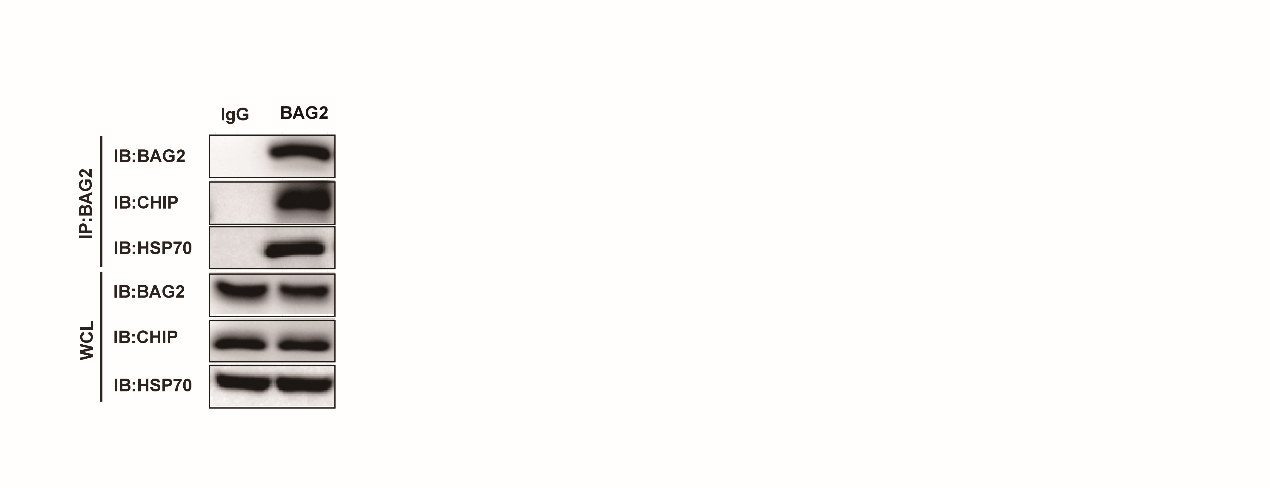


**Supplementary Figure 3.** Co-immunoprecipitation assays of BAG2, CHIP, or HSP70 co-expressed in AGS cells. IP, immunoprecipitation; WCL, whole-cell lysates.


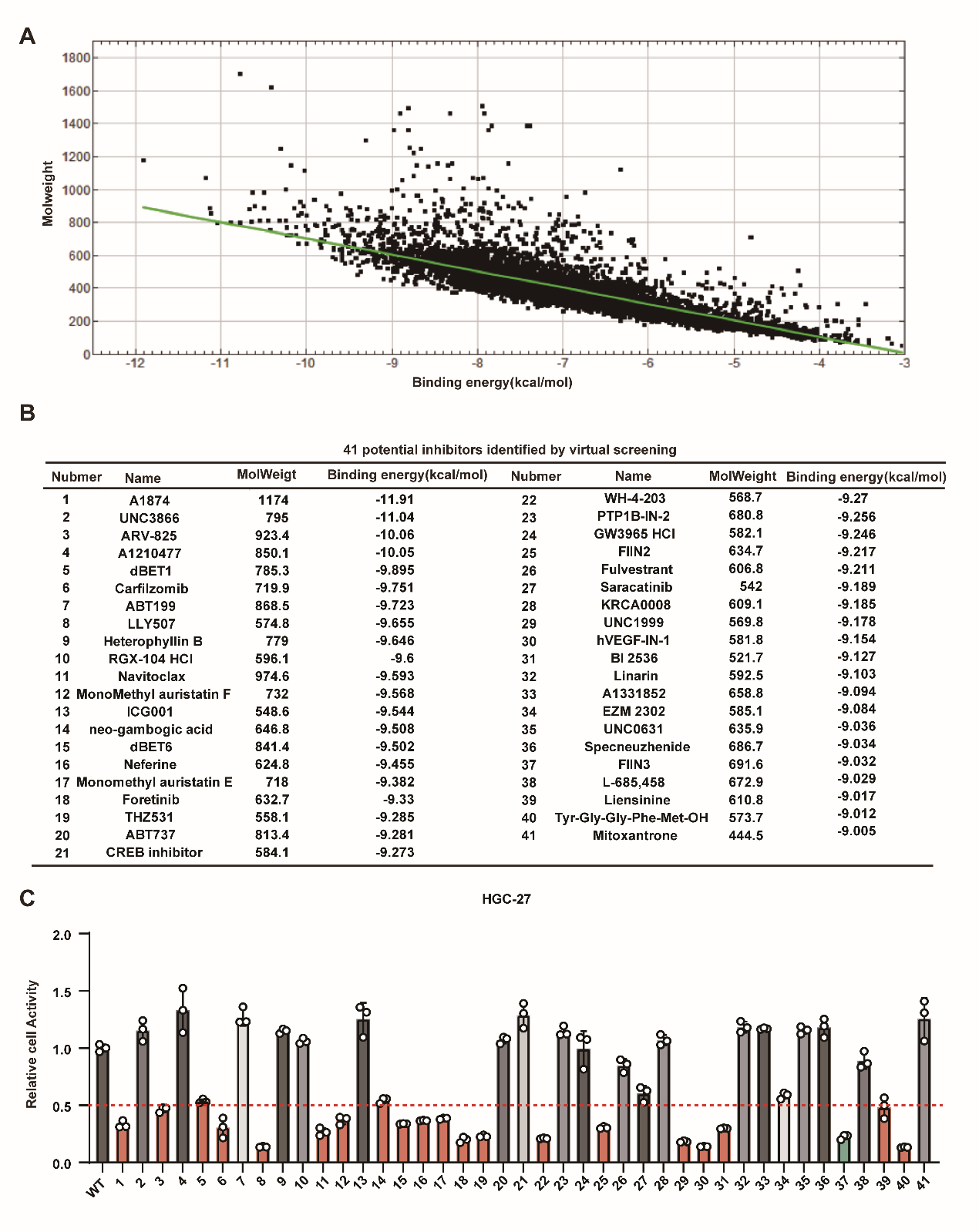


**Supplementary Figure 4.** Docking scoring of the top 41 small molecule compounds and the effects on HGC-27 cell viability were studied. **(A)** Docking score of 17 675 small molecules with CHIP using virtual screening. **(B)** Detailed docking scoring of top 41 small molecule compounds. **(C)** Effect of the top 41 small molecules on the activity of the GC cell line HGC-27.


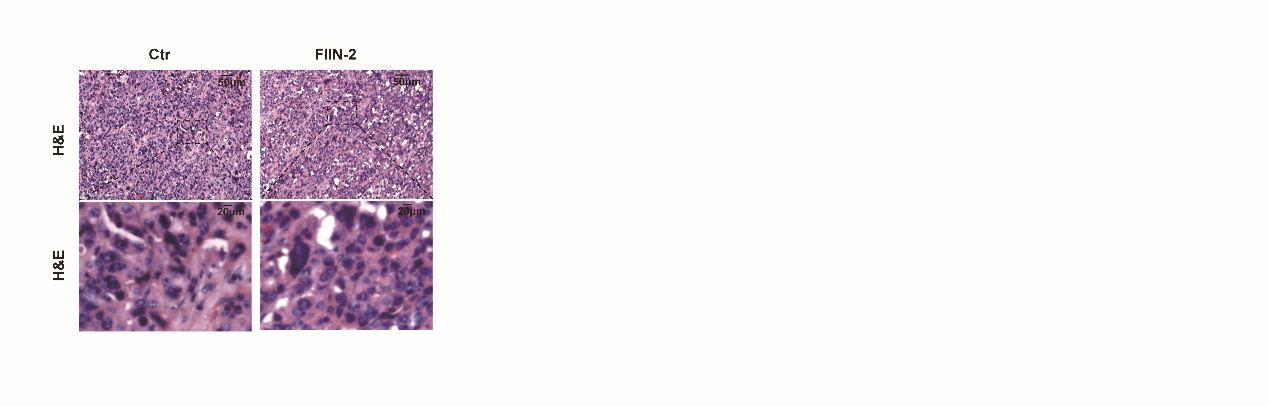


**Supplementary Figure 5.** Representative images of hematoxylin–eosin (H&E) in mice xenograft tumours treated with FIIN-2.


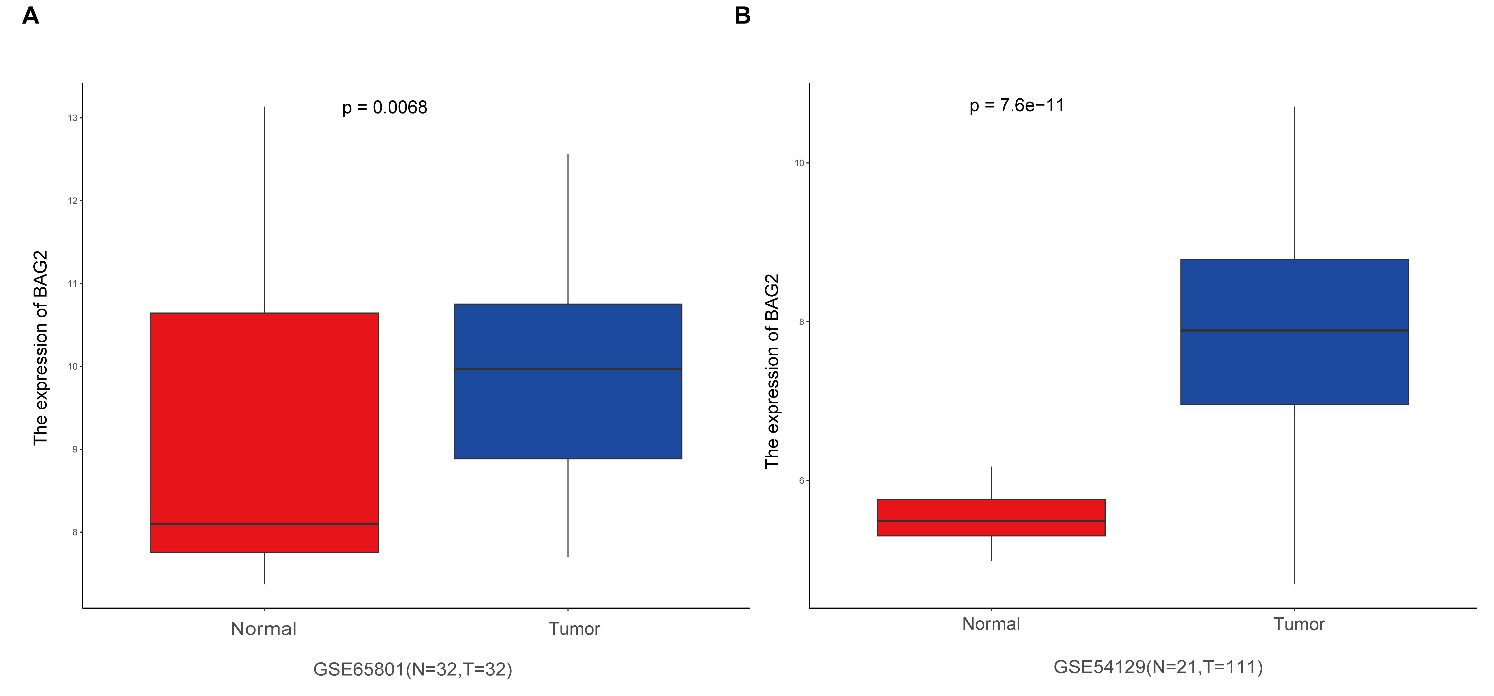


**Supplementary Figure 6.** The expression of BAG2 in gastric cancer was verified using the GEO database.
